# Bladder cancer survival in patients with *NOD2* or *CDKN2A* variants

**DOI:** 10.18632/oncotarget.28226

**Published:** 2022-04-22

**Authors:** Elżbieta Złowocka-Perłowska, Thierry van de Wetering, Aleksandra Tołoczko-Grabarek, Rodney J. Scott, Jan Lubiński

**Affiliations:** ^1^Department of Genetics and Pathology, International Hereditary Cancer Center, Pomeranian Medical University, Szczecin, Poland; ^2^Department of Clinical and Molecular Biochemistry, Pomeranian Medical University, Szczecin, Poland; ^3^School of Biomedical Sciences and Pharmacy, Centre for Information-Based Medicine, Hunter Medical Research Institute, University of Newcastle, Newcastle, NSW 2305, Australia; ^4^Division of Molecular Medicine, Pathology North, NSW Pathology, Newcastle, NSW 2305, Australia; ^*^These authors contributed equally to this work

**Keywords:** bladder cancer, kidney cancer, *NOD2*, *CDKN2A*, survival

## Abstract

Purpose: The association between the *NOD2* c.3020insC allele and *CDKN2A* missense variant c.442G>A (p.P.A148T) and survival of patients with bladder or kidney cancer remains controversial.

Materials and Methods: We compared the allele frequencies of *NOD2* c.3020insC and *CDKN2A* p.A148T allele in 706 patients with bladder cancer, 410 cases with kidney cancer against two control groups. The Cox proportional hazards model was used to determine whether there were any survival differences between carriers of the *NOD2* c.3020insC or the *CDKN2A* p.A148T variant.

Results: Among the three patient subgroups: patients under 60 years of age, non-smokers and a third with histological features of low grade noninvasive papillary bladder cancer, we observed that the c.3020insC allele had a nominal statistically significant effect on survival. We also observed that the *NOD2* c.3020insC variant was more frequent in patients with bladder cancer aged between 51 and 60 years.

There was some nominal evidence that the *CDKN2A* p.A148T polymorphism reduced survival in the subgroup of bladder cancer patients under 60 years of age. We observed that in kidney cancer patients, the incidence of the *NOD2* variant appeared to be lower in the group aged between 60 and 70 years, however, this was not statistically significant. In addition, in patients with histological features of grade III chromophobic kidney cancer, the c.3020insC allele also appeared to be over-represented but this too was not statistically significant.

Conclusion: We have shown that the *NOD2* c.3020insC allele and the *CDKN2A* p.A148T polymorphism does not play a role in the survival of patients with bladder cancer.

## INTRODUCTION

The multi-organ influence of variants in *NOD2* and *CDKN2A* are associated with the risk of cancers occurring in the colon, ovary, breast, lung, larynx, Non-Hodgkin lymphoma and melanoma [1–16]. The *NOD2* c.3020insC allele has been shown to occur in 7.3% of the Polish population [1] and could therefore be considered a common genetic risk factor for cancer. In 2005, we investigated 172 bladder and 245 kidney cancer patients [1] and observed nominal evidence for a relationship between the c.3020insC variant and bladder cancer (10.5%; odds ratio OR = 1.5; *p* = 0.13). We also reported a significantly lower frequency of the c.3020insC allele among renal cancer patients (3.2%; odds ratio OR = 0.4; *p* = 0.02) [6]. The *CDKN2A* p.A148T polymorphism is found in 3.5% of the Polish population [2]. In 2006 we determined the frequency of the *CDKN2A* p.A148T polymorphism in 223 patients with urothelial bladder cancer and 245 patients with kidney cancer [2]. The results of this study revealed the frequency of p. A148T to be similar in bladder or kidney cancer patients compared to controls. To ensure that our original findings were not erroneous and indeed represented the actual frequency found in bladder and kidney cancer patients, we re-evaluated the frequency of the *NOD2* c.3020insC allele and the p.A148T polymorphism in *CDKN2A* among 706 bladder cancer and 410 kidney cancer patients. Due to the size of the patient cohorts we were also able to evaluate the impact of these variants on survival of both the bladder and kidney cancer patients. To our knowledge, this is the first larger-scale study describing the clinical characteristics and survival of bladder and kidney cancer patients that is associated with the *NOD2* c.3020insC allele and the *CDKN2A* p.A148T polymorphism in Poland.

## RESULTS

### Bladder cancer

#### NOD2

The *NOD2* c.3020insC allele analysis was performed in 706 bladder cancer patients, the characteristics of the study population of bladder cancer are shown in Table 1. The frequency of the c.3020insC allele in the consecutive series of bladder cancer patients was significantly different compared to the control population, the characteristics of the patients with and without the c.c.3020insC allele are presented in Table 2, 63 (8.9%) patients carried the *NOD2* variant (OR = 1.4; 95% CI 1.01–1.90; *p* = 0.04).

**Table 1:** Characteristics of the study population of bladder cancer (*n* = 706)

Gender	
Male	540
Female	166
Age, mean (range)	79 (25–91)
≤60	222
>61	484
Smoking status	
Yes	461 (65%)
No	80 (11%)
Missing	165
Histological features	
Noninvasive papillary	416 (59%)
Low grade^1^	250 (35%)
High grade^2^	166 (24%)
Invasive	290 (41%)
Low	10 (1%)
High	280 (40%)
Stage	
Ta	415 (59%)
T1	118 (17%)
T2	84 (12%)
T3	60 (8%)
T4	29 (4%)
Vital status	
Alive	212 (30%)
Dead	494 (70%)

^1^low grade–GI. ^2^high grade-GII and GII.

**Table 2:** Clinical characteristics of bladder cancers patients harboring 3020insC allele in *NOD2* gene

	Patients with 3020insC allele (63)	Patients with no 3020insC allele (643)	*p*-value^*^	Odds ratio (CI)
Age of diagnosis (yr)				
≤50	3	55	0.42	0.5 0.16–1.76
51–60	23	141	0.01	2.0 1.18–3.53
61–70	14	212	0.10	0.6 0.31–1.07
>71	23	235	1.00	1.0 0.58–1.70
Mean	66	79		
Family history Positive (+)	3	23	0.90	1.3 0.39–4.62
Smoking				
no	10/49 (20)	70/492 (14)	0.30	1.5 0.73–3.24
≤20 packyears	11/49 (23)	123/492 (25)	0.80	0.9 0.43–1.75
>21	28/49 (57)	299/492 (61)	0.70	0.8 0.47–1.55
Histological features				
Noninvasive Papillary				
Low grade	24/40 (60)	226/376 (60)	1.00	1.0 0.51–1.94
High grade	16/40 (40)	150/376 (40)	1.00	1.0 0.51–1.95
Invasive				
Low grade	−	10/267 (4)	−	−
High grade	23/23 (100)	257/267 (96)	0.73	1.9 0.10–33.7
Stage				
Ta	40/63 (63)	375/643 (58)	0.50	1.2 0.72–2.12
T1	12/63 (19)	106/643 (16)	0.73	1.2 0.61–2.31
T2	7/63 (11)	77/643 (12)	1.00	0.9 0.40–2.09
T3	3/63 (5)	57/643 (9)	0.34	0.5 0.15–1.69
T4	1/63 (2)	28/643 (5)	0.47	0.3 0.04–2.65

^*^ *p*-values are calculated with respect to carriers of non-carriers.

The study subjects were followed from the date of diagnosis until death or February 2021 (a mean of 21 years). There were 40 deaths (63%) recorded in 63 *NOD2* c.3020insC carriers compared with 454 deaths (71%) in 643 non-carriers (HR = 0.94; 95% CI 0.67–1.31; *p* = 0.7).

**Figure 1:**
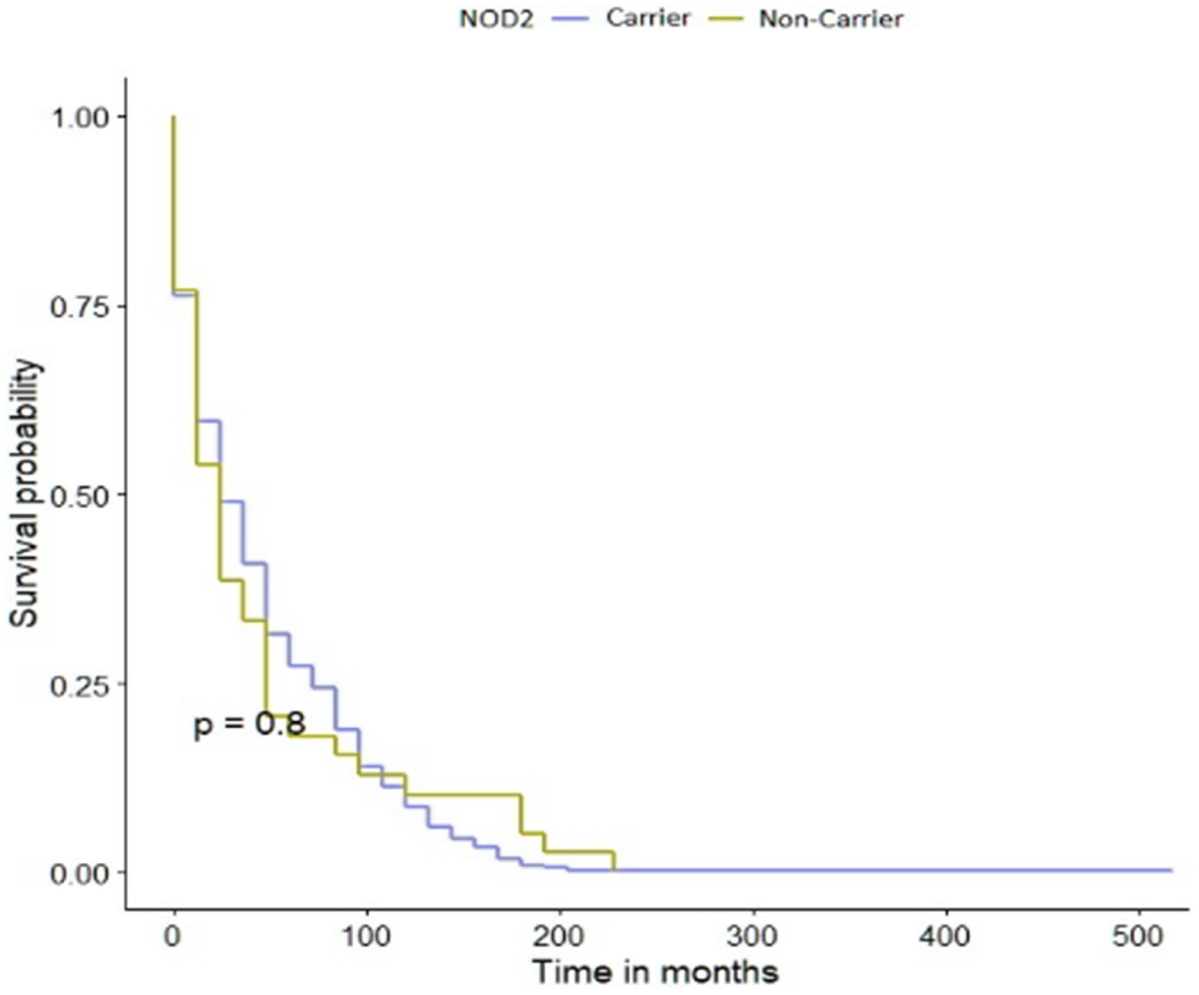
Kaplan-Meier survival curves of bladder cancer patients with *NOD2* variant and -negative sub-cohorts.

The *NOD2* c.3020insC allele did not play a significant role in the survival of patients with bladder cancer (Figure 1). The data was stratified for age, smoking status, cancer family history, sex and clinical characteristics. The median survival was 54 months for patients with the *NOD2* variant compared to 60 months for non-carriers (Table 3). Among patients with the c.3020insC allele the 10-year survival was 37% compared with 30% for non-carriers. After adjusting for age, smoking status, cancer family history, sex or clinical characteristics, the HR for mortality associated with bladder cancer and the *NOD2* variant are presented in Table 4. The HR for patients with positive cancer family history, invasive low grade or T4 couldn’t be calculated, due to low number of patients in those subgroups.

**Table 3:** Survival of patients with bladder cancer; by variant of *NOD2*

	Patients with *NOD2* variant (*n* = 63)	Patients with no variant in *NOD2* (*n* = 643)
Median follow-up (mo)	162	156
Proportion of deceased (%)	63	71
Median survival (mo)	54	60
5-Year survival (%)	50	50
10-Year survival (%)	37	30
HR	1.34	1.0
95% CI	0.89–2.01	−
*p*-value	0.2	−

Hazard ratio (HR), 95% confidence interval (CI), and *p*-values are calculated by cox ph test. Data was stratified for age, sex, clinical characteristics, smoking status and cancer family history.

**Table 4:** The HR for mortality associated with bladder cancer and the *NOD2* c.3020insC variant

	HR	95% CI	*p*-value
patients younger than 60 years old	2.14	0.89–5.16	0.0090
patients older than 60 years old	1.13	0.70–1.84	0.6
non-smoking patients	4.94	1.13–21.5	0.033
smoking patients	1.06	0.46–2.43	0.9
patients with no cancer family history	1.26	0.83–1.91	0.3
females	1.17	0.71–1.93	0.5
males	1.69	0.74–3.83	0.2
patients with noninvasive papillary low grade	3.11	1.38–7.01	0.006
patients with noninvasive papillary high grade	0.88	0.39–1.99	0.8
patients with invasive high grade	1.09	0.58–2.07	0.8
patients with Ta	1.45	0.84–2.51	0.2

#### CDKN2A

Of the 706 bladder cancer patients enrolled in the study, 37 (5.2%) carried a p.A148T variant in *CDKN2A* (OR = 1.5; 95% CI 1.04–2.24; *p* = 0.04). The characteristics of the study population are shown in Table 1. The characteristics of the patients with and without the polymorphism are presented in Table 5.

**Table 5:** Clinical characteristics of bladder cancers patients harboring A148T allele in *CDKN2A* gene

	Patients with 3020insC allele (37)	Patients with no 3020insC allele (669)	*p*-value^*^	Odds ratio (CI)
Age of diagnosis (yr)				
≤50	4	54	0.78	1.4 0.47–4.04
51–60	9	155	0.87	1.1 0.49–2.38
61–70	11	215v	0.90	0.9 0.43–1.84
>71	13	245	1.00	0.9 0.56–1.87
Mean	63	79		
Family history Positive (+)	1	25	0.74	0.7 0.09–5.43
Smoking				
no	4/28 (14)	76/513 (15)	0.93	1.0 0.32–2.84
≤20 packyears	7/28 (25)	166/513 (32)	0.54	0.7 0.29–1.67
>21	17/28 (61)	271/513 (53)	0.53	1.4 0.63–3.00
Histological features				
Noninvasive Papillary				
Low grade	14/25 (56)	236/391 (60)	0.80	0.8 0.37–1.89
High grade	11/25 (44)	155/391 (40)	0.80	1.2 0.53–2.70
Invasive				
Low grade	−	10/278 (36)	−	−
High grade	12/12 (100)	268/278 (96)	0.50	1.0 0.05–17.6
Stage				
Ta	25/37 (68)	390/669 (58)	0.34	1.5 0.73–3.01
T1	3/37 (8)	115/669 (17)	0.22	0.4 0.12–1.40
T2	5/37 (13)	79/669 (12)	0.95	1.1 0.44–3.08
T3	1/37 (3)	59/669 (9)	0.31	0.3 0.04–2.13
T4	3/37 (8)	26/669 (4)	0.40	2.2 0.63–7.57

^*^ *p*-values are calculated with respect to carriers of non-carriers.

**Figure 2:**
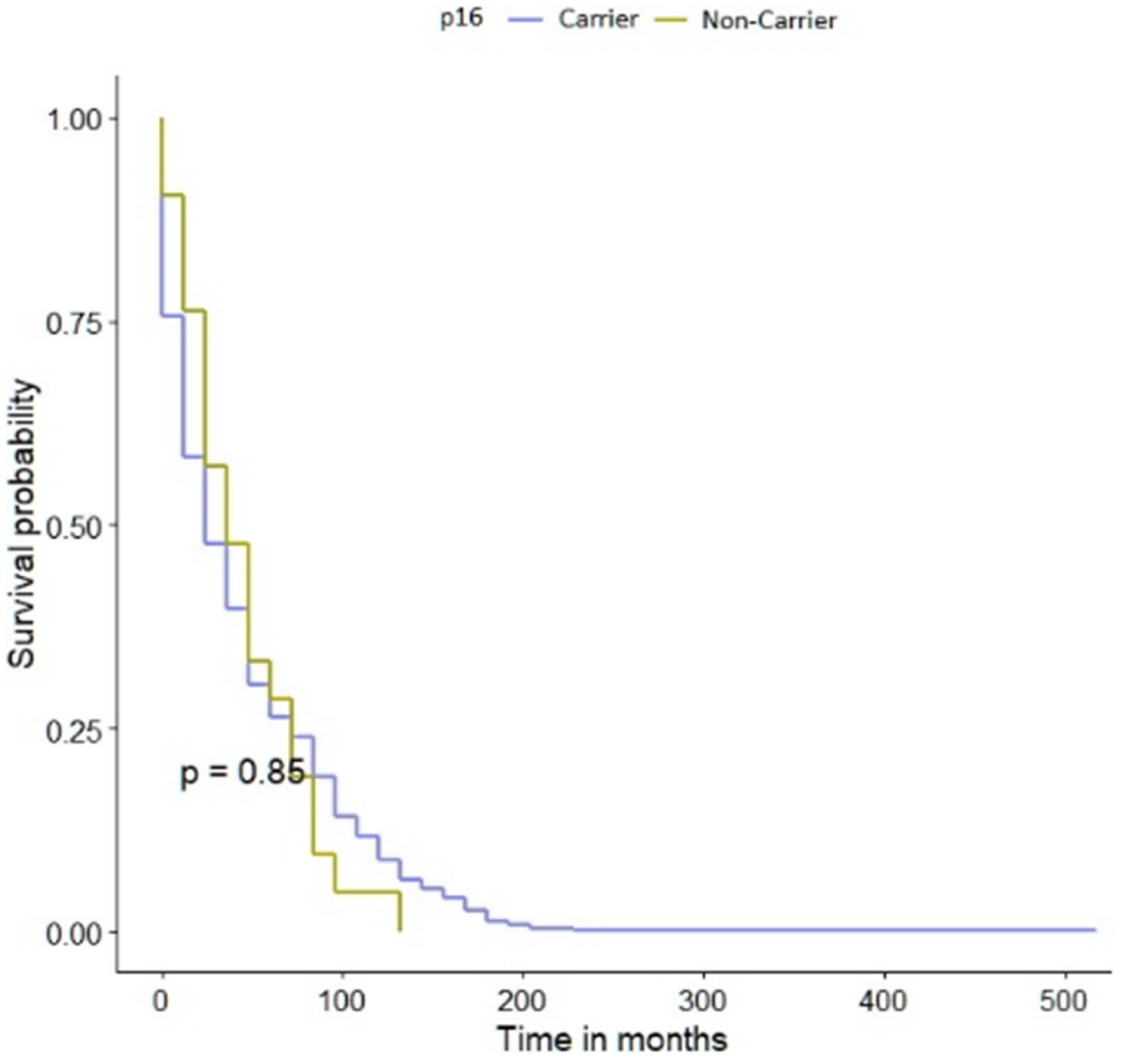
Kaplan-Meier survival curves of bladder cancer patients with *CDKN2A* polymorphism and -negative sub-cohorts.

The mean follow-up time was 35 years. There were 23 deaths (62%) in 37 p.A148T carriers versus 471 deaths (70%) in 669 non-carriers (HR = 1.10; 95% CI 0.73–1.68; *p* = 0.6). The p.A148T allele in *CDKN2A* gene did not affect the survival of patients with bladder cancer Figure 2. We observed a possible decrease in survival in the subgroup of patients younger than 61 years old carrying the p.A148T variant but this was not statistically significant. The data was stratified for age, smoking status, cancer family history, sex and clinical characteristics. The median survival of patients with *CDKN2A* polymorphism was the same as for non-carriers, namely 156 months (Table 6). In patients carrying the p.A148T allele the 10-year survival was 27% compared with 31% for non-carriers. After adjusting for age, smoking status, cancer family history, sex or clinical characteristics, the HR for mortality associated with bladder cancer and the *CDKN2A* variant are presented in Table 7. The HR for patients with positive cancer family history, T1, T3 or invasive low grade disease couldn’t be calculated, due to the low number of patients in those subgroups.

**Table 6:** Survival of patients with bladder cancer; by variant of *CDKN2A*

	Patients with *CDKN2A* variant (*n* = 37)	Patients with no variant in *CDKN2A* (*n* = 669)
Median follow-up (mo)	156	156
Proportion of deceased (%)	62	70
Median survival (mo)	72	48
5-Year survival (%)	54	50
10-Year survival (%)	27	31
HR	1.38	1.0
95% CI	0.83–2.29	−
*p*-value	0.2	−

Hazard ratio (HR), 95% confidence interval (CI), and *p*-values are calculated by cox ph test. Data was stratified for age, sex, clinical characteristics, smoking status and cancer family history.

**Table 7:** The HR for mortality associated with bladder cancer and the *CDKN2A* p.A148T variant

	HR	95% CI	*p*-value
patients younger than 60 years old	2.54	0.89–7.26	0.082
patients older than 60 years old	1.10	0.59–2.04	0.8
non-smoking patients	0.28	0.06–1.36	0.12
smoking patients	1.87	0.53–6.65	0.3
patients with no cancer family history	1.31	0.79–2.19	0.3
females	1.46	0.84–2.53	0.2
males	0.72	0.15–3.36	0.7
patients with noninvasive papillary low grade	1.15	1.50–2.64	0.7
patients with noninvasive papillary high grade	1.38	0.47–4.10	0.6
patients with invasive high grade	1.61	0.70–3.69	0.3
patients with Ta	1.21	0.63–2.31	0.6
patients with T2	1.09	0.42–2.80	0.9
patients with T4	5.29	0.32–86.9	0.2

### Kidney cancer

#### NOD2

The *NOD2* c.3020insC variant was detected in 7,3% of the kidney cancer patients, a similar frequency as found in the Polish population. The characteristics of the study population are shown in Table 8 both with and without the variant are presented in Table 9. A total of 30 patients carried the *NOD2* c.3020insC variant (OR = 1.1; 95% CI 0.74–1.69; *p* = 0.7) and was found in 20 affected men (5%) and 10 women (2.5%). The *NOD2* variant was not observed in familial cases with bladder cancer nor in first- and/or second-degree relatives. Sixteen cancer patients who carried the *NOD2* variant had died by February 2021.

**Table 8:** Characteristics of the study population of kidney cancer (*n* = 410)

Sex	
Male	261
Female	149
Age, mean (range)	60 (21–81)
≤60	166
>61	244
Smoking status	
Yes	162 (39%)
No	91 (22%)
Missing	157
Histological features	
Clarocellulare	
GI^*^	59 (14%)
GII^*^	159 (39%)
GIII^*^	105 (26%)
GIV^*^	29 (7%)
Chromophobe	
GI	16 (4%)
GII	5 (1%)
GIII	3 (1%)
Papillary	
GI	14 (3%)
GII	20 (5%)
GIII	−
Stage	
Ta	5 (2%)
T1	278 (68%)
T2	40 (8%)
T3	81 (20%)
T4	6 (2%)
Vital status	
Alive	278 (68%)
Dead	132 (32%)

^*^GI-GIV–Fuhrman Grade.

**Table 9:** Clinical characteristics of kidney cancers patients harboring 3020insC allele in *NOD2* gene

	Patients with 3020insC allele (30)	Patients with no 3020insC allele (380)	*p*-value*	Odds ratio (CI)
Age of diagnosis (yr)				
≤50	7	50	0.16	2.0 0.81–4.92
51-60	8	101	1.00	1.0 0.43−2.32
61-70	8	167	0.08	0.4 0.20−1.07
>71	7	62	0.30	1.5 0.64−3.79
Mean	61	60		
Family history Positive (+)	−	11	−	− −
Smoking				
no	4/19 (21)	87/234 (37)	0.20	0.4 0.14−1.40
≤20 packyears	8/19 (42)	95/234 (41)	1.00	1.0 0.41−2.74
>21	7/19 (37)	52/234 (22)	0.10	2.0 0.76−5.41
Histological features				
Clarocellulare				
GI	2/25 (8)	57/327 (17)	0.20	0.4 0.09−1.78
GII	11/25 (44)	148/327 (45)	1.00	0.9 0.42−2.15
GIII	8/25 (32)	97/327 (30)	0.80	1.1 0.46−2.67
GIV	4/25 (16)	25/327 (8)	0.10	2.3 0.73−7.22
Chromophobe				
GI	4/5 (80)	12/19 (63)	0.60	2.3 0.21−25.2
GII	−	5/19 (26)	−	− −
GIII	1/5 (20)	2/19 (11)	0.0006	34 2.43−474.86
Papillare				
GI	−	14/34 (41)	−	− −
GII	−	20/34 (59)	−	− −
GIII	−	−	−	− −
Stage				
Ta	−	5/380 (1)	−	− −
T1	18/30 (60)	260/380 (69)	0.41	0.7 0.32−1.48
T2	4/30 (13)	36/380 (9)	0.50	1.5 0.48−4.45
T3	8/30 (27)	73/380 (19)	0.30	1.5 0.65−3.57
T4	−	6/380 (2)	−	− −

^*^ *p*-values are calculated with respect to carriers of non-carriers.

#### CDKN2A

Of the kidney cancer patients enrolled in the study, fourteen (3.4%) carried a *CDKN2A* p.A148T polymorphism (OR = 0.9; 95% CI 0.55–1.70; *p* = 0.9). The characteristics of the study population are shown in Table 6 with and without the *CDKN2A* p.A148T variant are presented in Table 10. Eleven males (0.3%) and three females (0.7%) carried of the p.A148T variant. The p.A148T allele was seen in one family with kidney cancer present in first- and/or second-degree relatives. Seven patients with kidney cancer who carried the *CDKN2A* variant had died by February 2021.

**Table 10:** Clinical characteristics of kidney cancers patients harboring A148T allele in *CDKN2A* gene

	Patients with 3020insC allele (14)	Patients with no 3020insC allele (396)	*p*-value^*^	Odds ratio (CI)
Age of diagnosis (yr)				
≤50	1	56	0.70	0.4 0.05–3.64
51–60	3	106	0.76	0.7 0.20–2.72
61–70	6	169	1.00	1.0 0.34–2.95
>71	4	65	0.26	2.0 0.63–6.82
Mean	66	60		
Family history Positive (+)	1	10	0.32	2.9 0.35–24.9
Smoking				
no	5/9 (56)	86/244 (35)	0.28	2.3 0.60–8.78
≤20 packyears	3/9 (34)	100/244 (41)	0.74	0.7 0.17–2.94
>21	1/9 (10)	58/244 (24)	0.68	0.4 0.04–3.27
Histological features				
Clarocellulare				
GI	4/13 (31)	55/339 (16)	0.24	2.3 0.98–7.71
GII	6/13 (46)	153/339 (45)	1.00	1.0 0.34–3.16
GIII	3/13 (23)	102/339 (30)	0.76	0.7 0.18–2.58
GIV	−	29/339 (9)	−	− −
Chromophobe				
GI	−	16/23 (70)	−	− −
GII	1/1 (100)	4/23 (17)	0.20	13 0.45–374.8
GIII	−	3/23 (13)	−	− −
Papillare				
GI	−	14/34 (41)	−	− −
GII	−	20/34 (59)	−	− −
GIII	−	−	−	− −
Stage				
Ta	−	5/396 (1)	−	− −
T1	10/14 (71)	268/396 (68)	1.00	1.1 0.36–3.88
T2	1/14 (7)	39/396 (10)	1.00	0.7 0.08–5.53
T3	3/14 (22)	78/396 (20)	0.74	1.1 0.30–4.08
T4	−	6/396 (1)	−	− −

^*^ *p*-values are calculated with respect to carriers of non-carriers.

## DISCUSSION

We previously reported that the common founder mutation in *NOD2* may be more common in patients with bladder cancer and be associated with a reduced susceptibility to kidney cancer but these preliminary findings required further confirmation [1].

The study revealed that the presence of the *NOD2* c.3020insC variant and the CDKN2A p.A148T polymorphism did not influence bladder cancer survival. When examining subgroups of these patients, those at the age of 60 or younger, who did not smoke tobacco, and those with histological low grade noninvasive papillary bladder cancer there was nominal evidence that the *NOD2* c.3020insC variant effected survival.

When subdividing the patients by age to determine if there was any association with the age of disease onset we were unable to identify any statistically significant association between carriage of either of the two variants and the age of disease diagnosis. There also did not appear to be any relationship between either of the variants and the histological diagnosis of kidney cancer. Notwithstanding, we did identify borderline associations which require larger multi-center studies to determine more precisely whether there is a role of these two polymorphisms in disease risk and or pathology. To our knowledge no such large study examining the clinical characteristics and survival of Polish patients with bladder cancer and the presence of *NOD2* and *CDKN2A* variants has been reported to date.

The strengths of the current study are the number of bladder and kidney cancer patients, which is complemented by detailed participant data. Predictors of disease include sex, age, clinical characteristics, smoking status and cancer family history all of which were controlled for in our statistical analysis. The maximum period of follow-up was 210 months. Xu et al. reported a study of 1953 samples with information about *NOD2* from four The Cancer Genome Atlas (TCGA) databases in 2017 and showed that *NOD2* could be a predictor of survival of kidney cancer patients [15]. In the present study we were unable to perform a survival analysis for kidney cancer. Liu et al. in a meta-analysis of thirty case-control studies, showed that *NOD2* polymorphism was associated with an increased risk of gastric, breast, laryngeal, MALT lymphoma, lung and colorectal cancer [16]. In the review by Kutikhin (2011) the incidence of *NOD2* polymorphism in individual populations was compared [17]. In the Polish population, the *NOD2* c.3020insC variant has been identified in colorectal, early-onset laryngeal, lung, ductal breast cancer *in situ*, ovarian cancers [1, 3, 4, 6, 18]. In Poland, the association with the *NOD2* polymorphism and rectal [19]; gastric, prostate, thyroid, melanoma [1, 20]; pancreatic [1, 5]; breast [21] and ovarian [22] cancers was not confirmed. Many of the participants enrolled in these previous studies comprised only small number of patient groups, which is likely to have contributed to the variance in the associations observed.

Many studies have investigated the clinical-pathological and prognostic significance of *CDKN2A* gene in patients with bladder cancer. However due to differences in sample sizes, statistical analysis and the study populations, the results remain inconclusive and evidence-based confirmation from large-scale clinical trials is still lacking. To address these issues we conducted the current study on a large group of Polish patients. The meta-analysis described by Pan et al. examined 12 studies encompassing 975 patients with bladder cancer revealing no correlation between *CDKN2A* expression and cancer prognosis [23]. In contrast, Gan et al. undertook a meta-analysis of 37 studies (a total of 2246 patients) with bladder cancer which confirmed an association between *CDKN2A* expression with prognosis and clinical-pathological features in patients with bladder cancer. [24]. They suggested that low *CDKN2A* expression was correlated with worse prognosis for progression-free survival and recurrence-free survival in Ta–T1 bladder cancer. Sun et al. showed that mutation in *CDKN2A* plays an important role in kidney cancer metastasis [25]. In 2006 Dębniak et al. evaluated the association between eleven malignancies and the *CDKN2A* p.A148T polymorphism in the Polish population [2]. No statistically significant relationship was observed for bladder or kidney with *CDKN2A* p.A148T variant. The results reported herein, concur with these findings and we conclude that this variant is not associated with disease risk or outcome.

In summary, the results of this study indicate that neither the *NOD2* c.3020insC variant or the *CDKN2A* p.A148T polymorphism are associated with the survival of bladder cancer patients regardless of age, cancer family history, smoking status and sex. Thus, the *NOD2* c.3020insC or the *CDKN2A* p.A148T polymorphism cannot be added to the list of genes that are associated with an increased susceptibility to bladder or kidney cancer at this time.

## MATERIALS AND METHODS

### Study population

#### Patients

The unselected case group consisted of 706 urothelial bladder cancer patients (166 women, 540 men, mean age at diagnosis 79 years, range 25–91) and 410 kidney cancer cases (149 women, 261 men, mean age at diagnosis 60 years, range 21–81). All cancers were diagnosed at the Urology Hospital in Szczecin and the Genetic Outpatients Clinic between the years 2000–2018. A total of 1520 bladder cancers and 870 kidney cancers were identified during the study period. Of these, 1419 bladder patients and 835 kidney cancer patients accepted the study invitation. During the interview at the Genetic Outpatients Clinic, the study objectives were explained, informed consent was obtained, family history and smoking status recorded, genetic counseling provided and a blood sample taken for DNA analysis. The pathological diagnosis of bladder and kidney cancer was confirmed by biopsy at one central pathology laboratory in Szczecin, Poland. All cases were unselected for age, sex, clinical characteristics (stage: T, grade, histopathological tumor type), smoking and family history. Clinical data were collected from patient records. If there was no information on the stage, grade and histopathological type of the tumor the case was excluded. Of the 1419 patients with bladder and 835 with kidney cancer, clinical information was missing for 713 bladder and 425 kidney cases and these subjects were excluded from the study. In total, we recorded data from 706 patients with bladder and 410 with kidney cancer. Detailed information on smoking status (pack years) was available for a subgroup of 541 (77%) patients with bladder and 253 (62%) kidney cancer. A family history was taken at the Genetics Outpatients Clinic and a lifestyle questionnaire completed. A total of 26 patients were identified with a family history of at least 1 bladder cancer in their first or second degree relatives and 11 with a family history of at least 1 kidney cancer in their first or second degree relatives. The vital status and date of death of all of the cases were obtained from the Polish Ministry of the Interior and Administration in February 2021. In total, we received information about the deaths of 494 (70%) patients with bladder cancer and 132 (32%) with kidney cancer. All patients and control subjects are of European ancestry and are ethnic Poles. The study was approved by the Ethics Committee of Pomeranian Medical University in Szczecin.

#### Controls

We used two control groups. The first control group consisted of 2068 unselected, cancer-free individuals. These controls were selected to investigate the potential association between the *NOD2* c.3020insC allele and bladder and kidney cancer. The control participants were collected from 1648 adults from Szczecin who submitted blood for paternity testing and 420 lists of adult patients from three family doctors from Szczecin. This control group was described in detail elsewhere [3].

The second control group included 3000 unselected cancer-free individuals to estimate the association between the *CDKN2A* p.A148T variant and bladder and kidney cancer. This control group consisted of 2000 newborns from 10 hospitals all over Poland and 1000 adults from the Szczecin area not selected for family history. This control group was described in detail elsewhere [10].

The allele frequencies for the *NOD2* and *CDKN2A* variants in our control groups were not dependent on age or sex, and the estimates of the polymorphism frequency in both genes were similar in younger and in older control groups.

#### Methods

DNA was extracted from peripheral blood for all participants. The c.3020insC and the p.A148T variants were genotyped using a TaqMan assay (Life Technologies, Carlsbad, CA) employing a LightCycler Real-Time PCR 480 System (Roche Life Science, Mannheim, Germany). All mutations were confirmed by Sanger sequencing using a BigDye Terminator v3.1 Cycle Sequencing Kit (Life Technologies), according to the manufacturer’s protocol. In all reaction sets, positive and negative controls (including a reaction without DNA) were used.

### Statistical analysis

#### Survival analysis

In order to perform the analysis, patient survival was followed from the date of bladder or kidney cancer diagnosis to the date of death or February 2021. Death was established by linkage to the Polish Vital Statistics Registry. All subjects in the study were linked to the records of the Vital Statistics Registry using a unique eleven digit identification number (PESEL). Death was all-cause mortality as no specific cause of death was available. The median follow-up was 210 months.

Kaplan-Meier survival curves were constructed for the variant carriers and non-carriers of the two tumour groups and the control participants. The comparison of the survival curves was performed by log-rank test. Multivariable Cox regression analysis was performed on these patients. Covariates included age (≤60; >61 years), gender (females; males), clinical characteristics (stage: T, grade, histopathological type of cancer), smoking status (non-smoking; smoking) and family history of cancer (negative; positive).

Survival analysis was performed first on all subjects, and then on subgroups of individuals divided by: age, gender, clinical characteristics, smoking and family history.

Due to the findings that the *NOD2* c.3020insC allele and the *CDKN2A* p.A148T variant were not statistical significant in patients with renal cancer, we did not perform a survival analysis.

#### Power calculation

The statistical power for the case-control analysis for bladder cancer is approximately 70% with a two-sided confidence of 0.95, while for the renal cancer group we found the statistical power below 10%. In order to obtain at least 80% statistical power in this group, more than 322 000 subjects would have to be analysed, which is not possible in our region or indeed, Poland.

#### Odds ratios

The frequencies of the *NOD2* c.3020insC allele and the *CDKN2A* p.A148T variant were compared in bladder and kidney cancer patients against the control group. Odds ratios were generated from two-by-two tables and statistical significance was assessed using the Fisher exact test where appropriate. The odds ratios were used as estimates of relative risk and were adjusted for age, sex, clinical characteristics, smoking and family history by multiple logistic regression.

### Ethics approval and consent to participate

The study was approved by Ethics Committee of the Pomeranian Medical University in Szczecin, Poland. All participants gave informed written consent prior blood donating.
